# Molecular dynamics for linear polymer melts in bulk and confined systems under shear flow

**DOI:** 10.1038/s41598-017-08712-5

**Published:** 2017-08-21

**Authors:** Soowon Cho, Sohdam Jeong, Jun Mo Kim, Chunggi Baig

**Affiliations:** School of Energy and Chemical Engineering, Ulsan National Institute of Science and Technology (UNIST), UNIST-gil 50, Eonyang-eup, Ulju-gun, Ulsan, 689-798 South Korea

## Abstract

In this work, we analyzed the individual chain dynamics for linear polymer melts under shear flow for bulk and confined systems using atomistic nonequilibrium molecular dynamics simulations of unentangled (C_50_H_102_) and slightly entangled (C_178_H_358_) polyethylene melts. While a certain similarity appears for the bulk and confined systems for the dynamic mechanisms of polymer chains in response to the imposed flow field, the interfacial chain dynamics near the boundary solid walls in the confined system are significantly different from the corresponding bulk chain dynamics. Detailed molecular-level analysis of the individual chain motions in a wide range of flow strengths are carried out to characterize the intrinsic molecular mechanisms of the bulk and interfacial chains in three flow regimes (weak, intermediate, and strong). These mechanisms essentially underlie various macroscopic structural and rheological properties of polymer systems, such as the mean-square chain end-to-end distance, probability distribution of the chain end-to-end distance, viscosity, and the first normal stress coefficient. Further analysis based on the mesoscopic Brightness method provides additional structural information about the polymer chains in association with their molecular mechanisms.

## Introduction

Polymers undergo a variety of processing conditions in practical polymer processes, such as the plastic extrusion process. It is crucial to understand the structural and dynamical behaviors of polymer molecules under various external conditions to economically manufacture high-quality products in such processes. Accordingly, numerous experimental and theoretical research efforts have explored the fundamental aspects behind the macroscopic rheological responses of dense polymeric fluids^1, 2^, which have enormously advanced our knowledge and enabled us to predict the material properties of polymers under specific conditions in a variety of practical applications. However, many unresolved rheological issues remain (especially from the microscopic viewpoint) for polymeric materials in bulk or confined systems, e.g., fundamental mechanisms underlying stress overshoot, interfacial slip, and melt instability for polymer melts under shear flow^2–8^. To systematically control such rheological phenomena, it is essential to comprehend the intrinsic molecular dynamics of individual polymer chains separately in bulk and confined situations and how they compare to each other; such an understanding would greatly help to build general knowledge to accurately capture the physical aspects that underlie such complex macroscopic responses of polymer systems and tune the material properties in response to an arbitrary external flow field.

In this work, we performed an in-depth analysis on the fundamental molecular mechanisms and dynamic characteristics of bulk and confined polymer melts under shear flow using atomistic nonequilibrium molecular dynamics (NEMD) simulations of unentangled (C_50_H_102_) and weakly entangled (C_178_H_358_) linear polyethylene (PE) melts. This work is in addition to various advanced experimental^9–13^ and numerical^14–22^ studies to reveal the individual chain dynamics in polymer solutions or melts under an external flow field. This molecular-level information attained by directly tracking down individual chain motions is applied to understand the rheological behaviors of representative mesoscopic and macroscopic structural and dynamical properties in response to the applied flow field in a wide range of flow strengths. We analyze the similarities and differences between the bulk and confined melt systems in the characteristic molecular mechanisms and rheological responses under various flow regimes.

## Method

Canonical NEMD simulations of monodispersed unentangled (C_50_H_102_) and entangled (C_178_H_358_) linear PE melts for bulk and confined systems under shear flow were conducted using the *p*-SLLOD algorithm^23^ implemented with a Nosé–Hoover thermostat^24, 25^. For both bulk and confined systems, we employed the Siepmann-Karaboni-Smit united-atom model^26^ (wherein the original rigid bond was replaced by a flexible bond with a harmonic spring), which has been most commonly applied to the simulations of PE melts^14, 15, 17–19^. The set of evolution equations for each system^14, 19^ was numerically integrated with the reversible reference system propagator algorithm (*r*-RESPA)^27^ using multiple time scales: a short time scale (0.47 fs) for bonded (bond-stretching, bond-bending, and bond-torsional) interactions and a long time scale (2.35 fs) for the nonbonded Lennard-Jones interactions, thermostat, and external flow field. Both bulk and confined systems were subjected to a simple shear flow for which only the *xy*-component of the velocity gradient tensor was non-zero, with *x*, *y*, and *z* in Cartesian coordinates representing the flow, velocity gradient, and neutral directions, respectively. The system conditions for all bulk and confined PE melts studied in this work correspond to a constant temperature *T* = 450 K and pressure *P* = 1 atm. Specifically, for bulk systems, the simulations were executed at densities of *ρ* = 0.743 g/cm^3^ and *ρ* = 0.782 g/cm^3^ for the C_50_ PE melt and C_178_ PE melt, respectively. The simulation box dimensions for the bulk systems were set as (93.02 Å × 45.00 Å × 45.00 Å) (*x* × *y* × *z*) with a total of 120 molecules for the C_50_ PE melt, and [(65.89, 131.78, and 263.55) Å × 65.89 Å × 65.89 Å] (enlarged in the flow (*x*-)direction depending on the applied shear rate to avoid system-size effects) with a total of 54, 108, and 216 molecules, respectively, for the C_178_ PE melt. For confined systems where PE melts are confined by the two-layered rigid simple-cubic lattice walls in the velocity gradient (*y*-)direction, the simulations were carried out at *ρ* = 0.763 g/cm^3^ and *ρ* = 0.789 g/cm^3^ for the C_50_ PE melt and C_178_ PE melt, respectively. The walls were composed of 544 atoms with simulation box dimensions of (93.02 Å × 49.51 Å × 45.00 Å) for the C_50_ PE melt containing 120 molecules, and 676, 1352, and 2704 atoms with the box dimension of [(65.89, 131.78, and 263.55) Å × 70.51 Å × 65.89 Å] containing 54, 108, and 216 molecules, respectively, for the C_178_ PE melt. The lattice parameter of the simple cubic wall was set to *σ*_*w*_ = 5.227 Å (=1.33 *σ*_CH2_). The surface energy parameter of the wall atoms was set as *ɛ*_*w*_ */k*_*B*_ = 939 K ( = 20 *ε*_CH2_ */k*_*B*_), corresponding to a mica surface (~200–400 mJ/m^2^)^28, 29^. The wall atoms were fixed in their lattice sites during the simulations. For the confined systems, the shear flow field was generated by moving the upper wall at a constant velocity, *V*, in the flowing direction and fixing the bottom wall (the readers are referred to the Supplementary Information for further methodological details).

A wide range of flow strengths, from the linear to the highly nonlinear viscoelastic regimes, was applied to the bulk and confined C_50_ and C_178_ PE melts: 0.02 ≤ *Wi* ≤ 200 for C_50_ bulk PE, 0.05 ≤ *Wi* ≤ 500 for C_50_ confined PE, 0.39 ≤ *Wi* ≤ 7000 for C_178_ bulk PE, and 0.68 ≤ *Wi* ≤ 5600 for C_178_ confined PE. (The Weissenberg number (*Wi*) is defined as the product of the longest relaxation time (*τ*) of the system and the applied shear rate ($\dot{\gamma }$), $Wi\,\equiv \,\tau \dot{\gamma }$.) The characteristic relaxation time, *τ*, for each system evaluated by the integral below the stretched-exponential curve describing the decay of the time autocorrelation function of the unit chain end-to-end vector was *τ* = 0.5 ± 0.05 ns for C_50_ bulk PE, *τ* = 1.2 ± 0.1 ns for C_50_ confined PE, *τ* = 15.6 ± 1.0 ns for C_178_ bulk PE, and *τ* = 26.7 ± 2.0 ns for C_178_ confined PE work. (﻿Bef﻿ore collecting data, each system was fully equilibrated for a long time (i.e., several times longer than its longest relaxation time τ) at its target state point. After equilibration, the production run of more than 8–10 times longer than τ was carried out to evaluate statistically-reliable physical ﻿properties for each system; i.e., 5 ns and 15 ns for the C50 bulk and confined PE melts, respectively, and 100 ns and 200 ns for the C178 bulk and confined PE melts, respectively).

## Results and Discussion

**Figure 1:**
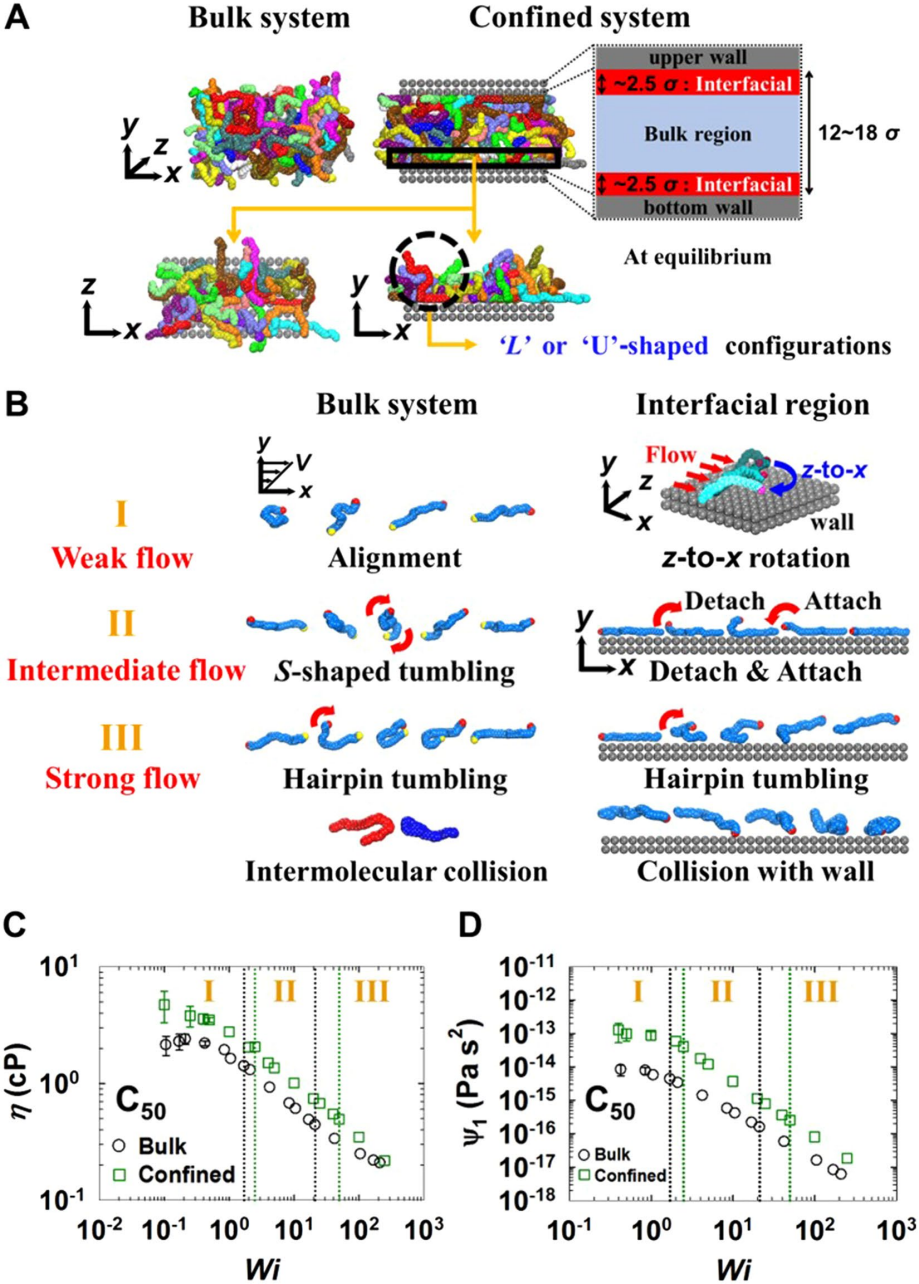
Characteristic molecular mechanisms and rheological properties. (**A**) Schematic illustration for the simulated bulk and confined polyethylene (PE) melt systems at equilibrium. For confined systems, the interfacial region is defined as the region located vertically within 2.5 *σ* (*σ* = 3.93 Å) of the wall surfaces. (**B**) Characteristic molecular dynamic mechanisms of polymer chains for the bulk system and the interfacial region of the confined system under shear in the three respective (weak, intermediate, and strong) flow regimes. Comparisons between the bulk and confined systems for (**C**) shear viscosity *η* and (**D**) the first normal stress coefficient (Ψ_1_) as a function of *Wi* for the C_50_ PE melt under shear flow. The vertical dotted lines distinguish the three characteristic flow regimes for bulk (black) and confined (green) systems. The three characteristic flow regimes are defined, based on the variation of the mean-square end-to-end distance for the bulk systems and the variation of the degree of slip (referring to ref. 19 for details) for the confined systems. The error bars are smaller than the size of the symbols, unless otherwise specified. See Supplementary Fig. 1 for the corresponding plot of the confined system with the *Wi* number based on the real shear rate accounting for a non-zero slip at the wall.

Figure 1A illustrates the bulk and confined systems studied in this work. For confined systems, if the center-of-mass position of a chain is located within 2.5 *σ* (*σ* = 3.93 Å) from the wall surfaces, it is considered as an interfacial chain. Under equilibrium conditions, in contrast to isotropic random-coil configurations displayed by chains in the bulk system, interfacial chains of confined systems are shown to possess mostly “*L*” or “U”-shaped (rather extended) configurations on the wall surfaces via the combined effects of the intramolecular chain conformational entropy and the favorable energetic interactions between the polymer and wall. That is, some parts of an interfacial chain are attached to the wall (i.e., adsorbed) and others are detached from the wall (i.e., non-adsorbed). While the adsorbed chain segments experience the wall friction through direct interactions with the wall, the non-adsorbed segments experience intermolecular interactions with nearby surrounding chains in the bulk region. This dual interaction induces distinctive molecular mechanisms for interfacial chains at a given flow strength under shear from those for chains in bulk system or bulk region in confined system.

First, for the bulk system (left panel of Fig. 1B), chains are mainly aligned to the flow (*x*-)direction at low shear rates without a significant structural deformation because the orientation is easier than stretching in response to the applied rotational shear field. At intermediate flow strengths, chains are substantially deformed (stretched) and nearly aligned to the flow direction, and furthermore begin to execute a whole-chain rotation and tumbling motion. In this flow regime, bulk chains mostly exhibit rather symmetrical *S*-shaped rotations and tumbling behaviors (Fig. 1B). This indicates that the head and tail portions of a chain almost equally (i.e., symmetrically) move in opposite directions relative to each other, as a result of the relative difference in their streaming velocity according to their different (higher and lower) positions in the velocity gradient direction of the applied shear field. In contrast, at high shear rates, chains mainly exhibit tumbling behaviors with hairpin-like configurations, rather than the symmetrical *S*-shaped configuration. This hairpin-like rotational characteristic occurs because a strong flow field does not allow a sufficient time for the head and tail portions of chains to symmetrically execute their respective movements during the rotational time span; i.e., either head or tail portion alone leads the overall chain tumbling motion quickly without waiting for the other portion to move in the opposite flowing direction.

In comparison, for confined system, chains near the wall exhibit distinctive characteristic molecular mechanisms with respect to the flow strength. In the weak flow regime, chains perform a *z*-to-*x* rotation (i.e., rotation from the *z*-direction to the *x*-direction) with still residing at the interface through the attractive polymer-wall interactions^19^. The alignment of interfacial chains with the flow (*x*-)direction via this in-plane chain rotation reduces the wall friction experienced by the chains during their movement along the flow direction through a decrease in the effective collision area between the chain and wall^19^. In the intermediate flow regime, the majority of interfacial chains display the out-of-plane wagging mechanism^19^, wherein outer parts of interfacial chains exhibit a repetitive motion between the detachment from and attachment to the wall (leading to an “*L*”-shaped configuration in the *x*-*y* plane) via the competitive effects of the applied flow field (inducing detachment) and the attractive polymer-wall interaction (enhancing attachment). In this flow regime, chains outside the interfacial region are substantially deformed and aligned to the flow direction, which significantly affects the degree of interaction between interfacial and nearby surrounding bulk chains. With further increasing flow strength, interfacial chains experience strong dynamical collisions with the wall atoms, which gives rise to highly nonlinear rotational dynamics, i.e., irregular (chaotic) rotation and tumbling mechanisms at the wall, leading the interfacial chains to detach from the wall toward the bulk region^19^. Interfacial chains can only execute a hairpin-like tumbling motion while respecting the geometrical constraint imposed by the wall.

All these characteristic molecular mechanisms and dynamics underlie the macroscopic structural and dynamical properties for bulk and confined systems, respectively. Figure 1C presents the viscosity variation with respect to the *Wi* number for the bulk and confined C_50_ PE melt systems. Both bulk and confined PE melts exhibit a typical shear-thinning behavior, and the degrees of shear thinning are similar. However, the viscosity of the confined system is larger than that of the bulk system in the entire flow regime, which is consistent with experiments^9^. This is attributed to the high degree of momentum transfer via collisions (friction) between the interfacial chains and the wall. A similar behavior is shown for the first normal stress coefficient in Fig. 1D; this is again ascribed to the large contribution of interfacial chains to the overall system elasticity, arising from their highly oriented and stretched conformations.

**Figure 2:**
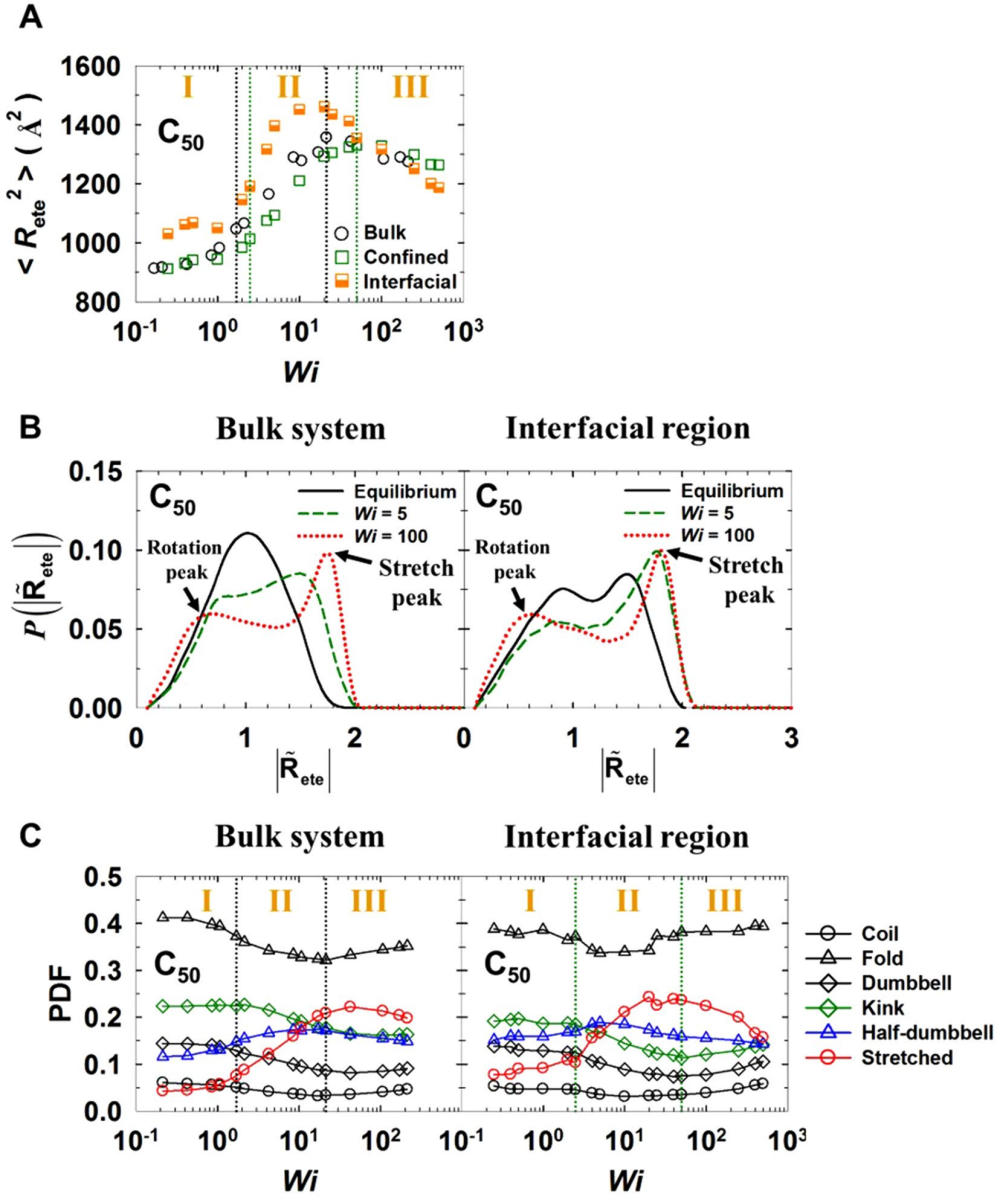
Comparison of the mesoscopic and macroscopic structural properties of the bulk and confined systems. (**A**) Mean-square chain end-to-end distance 〈*R*_ete_^2^〉 as a function of *Wi* for the bulk and confined C_50_ PE melt systems. “Interfacial” represents the corresponding result for only the interfacial chains in the confined system. The interfacial regions of both top and bottom walls for the confined systems are found to produce practically identical results (within the statistical uncertainties) for all the microscopic and macroscopic structural and dynamical properties, e.g., 〈Rete2〉 and its PDF, molecular conformations via the Brightness method, interfacial residence time, and so forth. The error bars are smaller than the size of the symbols unless otherwise specified. Comparison of the bulk system and the interfacial region in the confined system for the C_50_ PE melt for (**B**) the probability distribution function, ${P}(|{\tilde{{\bf{R}}}}_{{\rm{ete}}}|)$, of the chain end-to-end distance (normalized by the equilibrium value ($|{\tilde{{\bf{R}}}}_{{\rm{ete}}}|=|{{\bf{R}}}_{{\rm{ete}}}|/{|{{\bf{R}}}_{{\rm{ete}}}|}_{{\rm{eq}}}$) for each system), and (**C**) the probability distribution function (PDF) of the six representative mesoscale chain configurations (Coil, Fold, Kink, Dumbbell, Half-dumbbell, and Stretched) as a function of *Wi* computed by the Brightness method.

In Fig. 2A, we compare the mean-square chain end-to-end distances 〈*R*_ete_^2^〉 as a function of shear rate for the bulk and confined systems. For both the bulk and confined systems, the overall behavior of 〈*R*_ete_^2^〉 can be characterized by three distinct flow regimes. In the weak flow regime, 〈*R*_ete_^2^〉 displays a slight increase with shear rate, indicating that chains in this regime are mostly oriented to the flow direction without a significant structural deformation or stretch. In the intermediate regime, 〈*R*_ete_^2^〉 exhibits a dramatic increase with the applied flow strength and eventually reaches a maximum. With a further increase of the shear rate, 〈*R*_ete_^2^〉 shows a rather decreasing behavior, which was also observed in previous studies^14, 15, 18^ for bulk PE melts under shear flow. The decrease is ascribed to strong intermolecular collisions together with intense chain rotation and tumbling dynamics at high shear rates. However, we should keep in mind that the characteristic molecular mechanism behind the variation of 〈*R*_ete_^2^〉 for each flow regime is not the same for the bulk and interfacial chains.

The 〈*R*_ete_^2^〉 value is quantitatively quite similar for the bulk and confined systems with respect to *Wi* (a further quantitative similarity is seen for the confined system in terms of *Wi* number based on the real shear rate, which accounts for a non-zero slip at the wall (Supplementary Fig. 2)). However, the average value of 〈*R*_ete_^2^〉 for only the interfacial chains appears to be somewhat larger than that of the whole confined system, indicative of a larger deformation of the interfacial chains (in association with a higher degree of molecular interactions with the wall) via the favorable polymer-wall interactions. There is also a relatively steeper increase of 〈*R*_ete_^2^〉 in the intermediate flow regime and a larger decrease of 〈*R*_ete_^2^〉 in the strong flow regime for interfacial chains than that for the bulk system. This phenomenon can be understood based on the molecular mechanisms of interfacial chains described in Fig. 1B. At flow strengths greater than that in the weak flow regime (where the characteristic molecular mechanism is the *z*-to-*x* in-plane chain rotation without a substantial structural deformation, i.e., only a slight variation of 〈*R*_ete_^2^〉), the chains exhibit the out-of-plane wagging mechanism with highly deformed structures (i.e., a large variation of 〈*R*_ete_^2^〉) in response to the applied flow. The steep increase of 〈*R*_ete_^2^〉 for the interfacial chains in the intermediate flow regime is associated with the repetitive chain attachment-detachment mechanism, because the interfacial chains mostly tend to be aligned and stretched in the flow direction without executing rotational or tumbling dynamics. In the strong flow regime, interfacial chains exhibit a chaotic (irregular) rotation and tumbling mechanism with strong dynamic collisions with the wall atoms; this considerably reduces the stretched chain conformation to a rather compact structure, leading to a significant decrease of 〈*R*_ete_^2^〉 with increasing flow strength. These stronger variations of 〈*R*_ete_^2^〉 for interfacial chains can be further understood by analyzing the probability distribution function of the chain end-to-end distance (Fig. 2B). Compared to the chains in the bulk system, the interfacial chains in the confined system exhibit a more pronounced stretch peak in the intermediate flow regime and a more distinctive rotation peak in the strong flow regime. In addition, the region between the rotation and stretch peaks displays a more pronounced curvature for interfacial chains compared to that for the corresponding bulk system. The interfacial chains exhibit two distinct peaks, even at equilibrium, indicative of a certain amount of chains with a rather extended conformation because of the energetically favorable polymer-wall interaction.

Further detailed structural information for the polymer chains under shear flow can be obtained from the Brightness method^15, 30^, which categorizes the mesoscale chain structures into several configuration classes based on the monomer distribution along the chain. Figure 2C presents the probability distribution function (PDF) for six representative configurations (Coil, Fold, Kink, Dumbbell, Half-dumbbell, and Stretched) for the C_50_ PE melt system. (It is noted that the Brightness method focuses on the overall chain configuration or shape without regard to the actual chain size). We note that rather short C_50_ PE chains produce higher portions for the Fold configuration than the Coil configuration at equilibrium. At low shear rates, the portions of Half-dumbbell and Stretched configurations appear somewhat larger for interfacial chains than those for the bulk system, indicative of more extended conformations of interfacial chains via attractive polymer-wall interactions. More interesting features are exposed in the intermediate flow regime where, with increasing shear rate, (i) the Fold portion exhibits a gradual decrease for the bulk system but a small increase for interfacial chains, (ii) the Half-dumbbell portion exhibits a small increase for the bulk system but a small decrease for interfacial chains, and (iii) the Stretched portion is quite larger for interfacial chains than for the bulk system. These results can be understood by considering the characteristic molecular dynamics corresponding to the *S*-shaped tumbling mechanism for the bulk system and the *L*-shaped repetitive chain detachment-attachment for interfacial chains. In the strong flow regime, the stretched portion appears to decrease more significantly for interfacial chains compared to that for the bulk system, as associated with irregular (chaotic) chain rotation and tumbling mechanisms of interfacial chains. In addition, the dominant hairpin-like chain tumbling mechanism for the bulk system at high shear rates leads to a relatively larger increase in the Fold portion than that for the interfacial chains.

**Figure 3:**
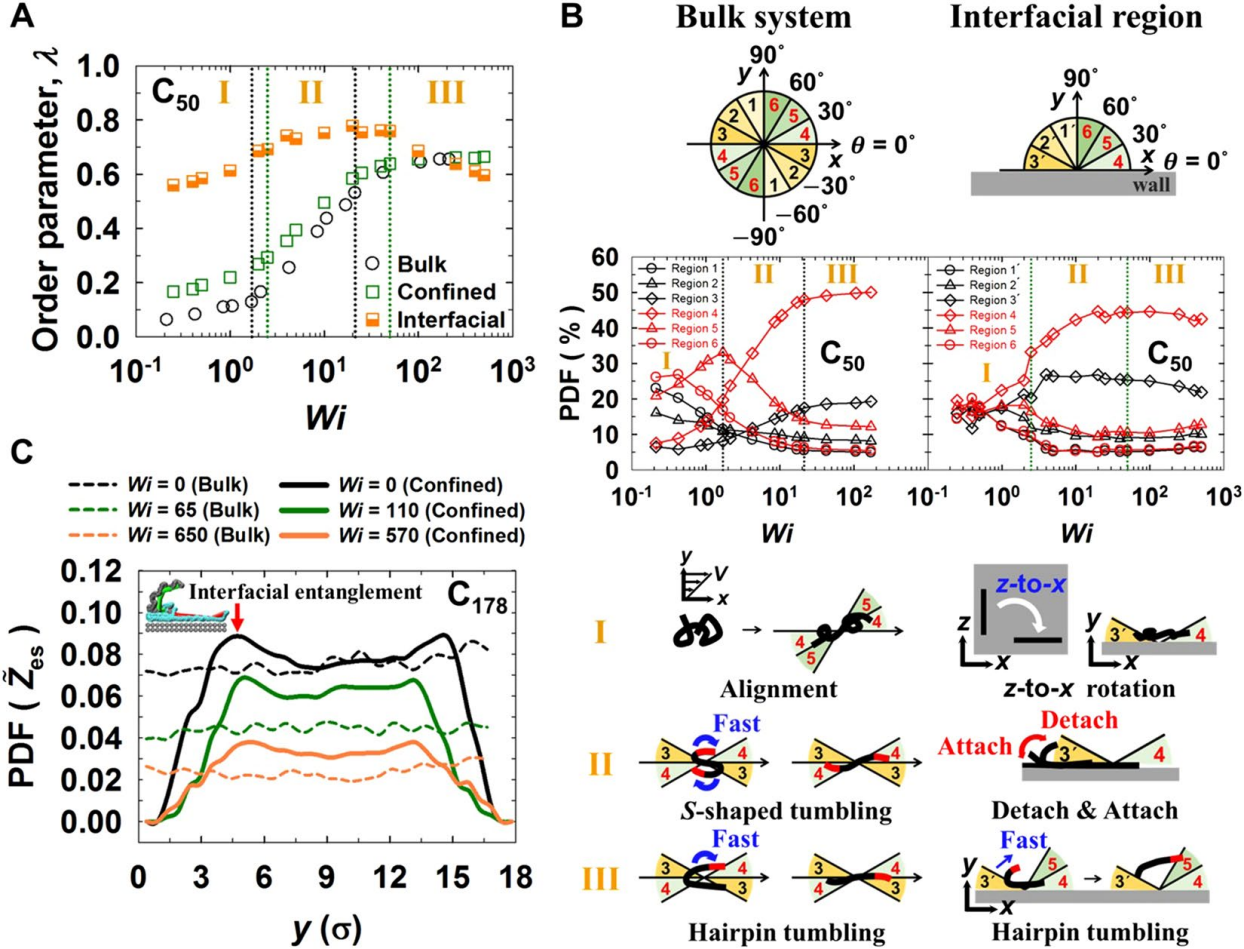
Comparisons of the order parameter, chain orientational distribution, and entanglement density distribution for the bulk and confined systems. (**A**) Chain order parameter *λ*, which corresponds to the largest eigenvalue of the order tensor ${\bf{S}}=\langle (3{\bf{uu}}-{\bf{I}})\rangle /2$, where **u** denotes the unit chain end-to-end vector and **I** denotes the second-rank unit tensor, as a function of *Wi* for the bulk and confined C_50_ PE melt systems. “Interfacial” represents the corresponding result for only the interfacial chains in the confined system. The error bars are smaller than the size of the symbols, unless otherwise specified. See Supplementary Fig. 3 for the corresponding plot of the confined system with *Wi* number based on the real shear rate accounting for a non-zero slip at the wall. (**B**) Probability distribution function (PDF) of the chain orientation angle (based on the chain end-to-end vector) with respect to the flow direction as a function of *Wi* with a schematic illustration of the molecular mechanisms in conjunction with the PDF in the three respective flow regimes. The total space is divided by six angular regions for the bulk system and the interfacial region of the confined system. (**C**) PDF of the local entanglement density (${\tilde{{Z}}}_{{\rm{es}}}={Z}_{{\rm{es}}}/{\sigma }^{3}$) along the velocity gradient (*y*-)direction for the entangled C_178_ PE melts of the bulk and confined systems. Note that *Wi* = 0 corresponds to the equilibrium condition, *Wi* = 65 for the bulk system and *Wi* = 110 for the confined system are in the intermediate flow regime, and *Wi* = 650 for bulk system and *Wi* *=* 570 for the confined system are in the strong flow regime.

Figure 3A presents the variation of the chain order parameter, *λ*, for the C_50_ PE melt with respect to the shear rate, which measures the degree of chain alignment in the flow direction in response to the applied field. The interfacial chains already exhibit a large degree of chain ordering in the weak flow regime, with a gradual increase of *λ* with the shear rate and almost saturation of chain ordering at the end. As such, the *λ*-value exhibits only a slight variation with shear rate in the intermediate flow regime. In contrast, the corresponding bulk system displays a rather small increase in *λ* with respect to the shear rate in the weak flow regime and a steep increase in the intermediate flow regime, followed by a plateau region in the strong flow regime. We note that qualitatively similar behavior is observed for the confined system when *λ* is calculated over all the chains of bulk and interfacial regions; thus, the order parameter averaged over the entire confined system (which might be the case in typical experiments) may provide erroneous structural information for interfacial chains. Furthermore, in contrast to the plateau values of *λ* for bulk chains in the strong flow regime, the *λ*-value for the interfacial chains decreases considerably with increasing shear rate in this regime. This phenomenon is directly associated with the irregular (chaotic) chain rotation and tumbling mechanisms via strong molecular collisions of interfacial chains with the wall.

Further information related to the chain orientation was obtained by investigating the PDF of the chain orientation angle with respect to the flow direction for the C_50_ PE system; for this purpose, six spatial orientation regions were chosen, as depicted in Fig. 3B. In the weak flow regime, with increasing shear rate, the PDFs for the bulk system show a decrease for Regions 1, 2, and 6, but a somewhat increase for Regions 4 and 5. As a noticeable difference between the bulk and confined systems, the PDFs for interfacial chains exhibit a significant increase for Region 3 and 4 compared to those of the corresponding bulk system; this result directly shows a significant chain alignment to the flow direction for the interfacial chains at low shear rates, which is consistent with the result for the order parameter in Fig. 3A. In the intermediate flow regime, the bulk system shows a steep increase in the PDF of Region 4 but a decrease in the PDF of Region 5 with increasing shear rate. In addition, there is a slight increase in the PDF of Region 3, which is associated with an increase in the degree of the chain tumbling dynamics. In contrast, the interfacial chains exhibit a less steep increase in the PDF of Region 4 and a nearly constant value for the PDF of Region 3 in this flow regime, as can be understood by considering the out-of-wagging (repetitive chain detachment-attachment) mechanism. In the strong flow regime, the bulk system exhibits a slight increasing behavior for the PDFs of Regions 3 and 4 and a slight decreasing (or a plateau) behavior for those of the other regions. In contrast, the interfacial chains exhibit somewhat opposite trends, which are directly related to the intense irregular (chaotic) chain rotation and tumbling mechanisms of interfacial chains on the wall (see Supplementary Fig. 4 for the average tumbling time of chains in the bulk and confined C_50_ PE melts).

Figure 3C presents the spatial distributions along the velocity gradient (*y*-)direction for the entanglement density ${\tilde{{Z}}}_{{\rm{es}}}$ for the entangled C_178_ PE melts, which were obtained from the topological analysis of the entanglement network of the system using the Z1-code^31, 32^. (The average number of kinks (intermolecular entanglements) per chain directly obtained from the Z1-code analysis for the C178PE melt system is approximately equal to 6, which is roughly two times larger than the number of entanglements based on the experimental plateau modulus. ﻿O﻿ur various analyses show that the rheological characteristics of entanglement network for the C178PE melt under shear in a wide range of flow strengths is qualitatively very similar to that of the longer C400 PE melt for both bulk and confined systems. We therefore ﻿consider the p﻿resent result for the rheological aspects of entanglement network for the C178 PE melt to be qualitatively valid for the practical entangled syst﻿ems﻿). In contrast to the homogeneous distributions of ${\tilde{{Z}}}_{{\rm{es}}}$ for the bulk system, ${\tilde{{Z}}}_{{\rm{es}}}$ for the confined system exhibits a distinctive shoulder around each of the interfacial regions at equilibrium. This is mainly associated with a relatively higher polymer density^19^ in the interfacial regions via the polymer-wall interaction. In addition, ${\tilde{{Z}}}_{{\rm{es}}}$ is enhanced by entanglements between the detached chain segments of the *L* or U-shaped interfacial chains and the nearby surrounding bulk chains. This feature can be associated with a relatively higher viscosity (and thus a higher viscous dissipation under flowing conditions) in the region near the interface. At high shear rates, the ${\tilde{{Z}}}_{{\rm{es}}}$-value decreases throughout the confined system due to disentanglement between chains via chain alignment and stretching. Further, the distribution becomes flattened with less pronounced shoulders with their positions appearing somewhat shifted toward the system center. This phenomenon can be understood by considering the frequent movement of interfacial chains into the bulk region and mixing with bulk chains via strong dynamical collisions with the wall at high flow strengths.

## Conclusion

Through a detailed analysis of individual chain dynamics using atomistic NEMD simulations for unentangled (C_50_H_102_) and weakly entangled (C_178_H_358_) linear PE melts under shear flow in bulk and confined systems, we revealed and contrasted the characteristic molecular mechanisms with respect to the applied flow strength between the bulk and interfacial chains. This molecular dynamic information is very useful in understanding the structural and rheological behavior of bulk and confined systems under shear as a function of flow strength, and would be further beneficial for building our general knowledge of predicting and controlling the material properties of the polymer under various flow conditions. The main features identified in this study are summarized below.

- Under equilibrium conditions, while polymer chains in the bulk system display random-coil configurations, the interfacial chains of the confined system possess “*L*” or “U”-shaped configurations on the wall via the combined effects of the intramolecular entropy and the attractive polymer-wall interaction. The detached chain segments of the “*L*” or “U”-shaped interfacial chains make entanglements with nearby surrounding bulk chains, enhancing the degree of chain entanglement (and thus the viscosity) around the interfacial regions.
- In the weak flow regime, while both bulk and interfacial chains are aligned to the flow (*x*-)direction without a significant structural deformation, the interfacial chains of the confined system perform the *z*-to-*x* in-plane rotation at the wall. In addition, compared to the bulk chains, the interfacial chains achieve a much larger degree of chain ordering in the weak flow regime.
- In the intermediate flow regime, both bulk and interfacial chains become nearly aligned to the flow direction with a highly deformed (extended) structure; however, in comparison to bulk chains, interfacial chains display a considerably steeper increase of 〈*R*_ete_^2^〉 with respect to the shear rate and a more pronounced stretch peak in ${P}(|{\tilde{{\bf{R}}}}_{{\rm{ete}}}|)$. As regards the characteristic molecular mechanism, the interfacial chains reveal the *L*-shaped out-of-plane wagging (repetitive chain detachment-attachment) mechanism while chains in the bulk system exhibit a rather symmetrical *S*-shaped rotation and tumbling behavior. These distinct dynamic characteristics between the bulk and interfacial chains lead to significantly different results for the probability distribution with respect to the mesoscopic chain configurations in the Brightness method and chain orientation angle as a function of the flow strength in this regime.
- In the strong flow regime, both the bulk and interfacial chains exhibit chain end-over-end tumbling behaviors. Specifically, bulk chains exhibit a tumbling behavior mainly with a hairpin-like configuration instead of the symmetrical *S*-shaped one. In comparison, interfacial chains exhibit highly nonlinear rotational dynamics, such as irregular (chaotic) rotation and tumbling (strictly with a hairpin-like configuration) mechanisms at the wall, via strong dynamical collisions with the wall. This interfacial dynamics results in (i) a significant decrease of 〈*R*_ete_^2^〉, (ii) distinctive rotation peak in ${P}(|{\tilde{{\bf{R}}}}_{{\rm{ete}}}|)$, and (iii) distinct behavior for the probability distribution of the mesoscopic chain configurations in the Brightness method, with respect to the applied flow strength in this regime. In addition, such strong collisions lead the chains to frequently move out of the interfacial region toward the bulk region.

The findings in this study should be carefully taken into account in theoretical modeling. For example, a naïve use of the order parameter and the mean-square chain end-to-end distance averaged over the whole system may lead to erroneous predictions of rheological properties (e.g., stress, anisotropic diffusion, flow birefringence) for confined systems, since the intrinsic structural and dynamical behaviors of the bulk and interfacial chains in response to the applied flow are quite dissimilar. In addition, an adequate description for the flow-induced crystallization of confined systems would require combined information of the distinct characteristic molecular dynamics in each flow regime for both the bulk and interfacial chains. Considering the rapid advance in experimental technique (e.g., fluorescent video microscopy^10–13^), we also expect the present findings to be potentially useful in the experimental analysis and practical applications of bulk and confined dense polymeric materials undergoing shear flow in the near future.

## Electronic supplementary material

Supplementary Information
