# Supplementary material for: Molecular dynamics for linear polymer melts in bulk and confined systems under shear flow
Source: Sci Rep. 2017 Aug 21;7:9004. doi: 10.1038/s41598-017-08712-5 (PMC5567124; doi:10.1038/s41598-017-08712-5)
Supplement: Supplementary file 1 — Supplementary Information [file 41598_2017_8712_MOESM1_ESM.pdf]

Supplementary Information

Molecular dynamics for linear polymer melts in bulk and confined systems under shear flow

Soowon Cho, Sohdam Jeong, Jun Mo Kim*, and Chunggi Baig*

School of Energy and Chemical Engineering, Ulsan National Institute of Science and Technology(UNIST), UNIST-gil 50, Eonyang-eup, Ulju-gun, Ulsan, 689-798, South Korea

*Authors to whom correspondence should be addressed

Email: junmokim@unist.ac.kr, cbaig@unist.ac.kr

Phone: +82-52-217-2538

Fax: +82-52-217-2649

The Supplementary Information includes:

- **Simulation Methodology**
- **Figures S1-S4**

Simulation Methodology

Canonical NEMD simulations of unentangled ($C_{50}H_{102}$) and entangled ($C_{178}H_{358}$) linear polyethylene melts for bulk and confined systems were carried out using *p*-SLLOD equations of motion under shear[23] with the Nosé–Hoover thermostat[24,25]. The modified *p*-SLLOD equations of motion with a Nosé-Hoover thermostat for confined systems have the following expressions:

$$\dot{\mathbf{r}}_i = \frac{\mathbf{p}_i}{m_i} + \mathbf{r}_i \cdot \nabla\mathbf{u},$$

$$\dot{\mathbf{p}}_i = \mathbf{F}_i(\mathbf{r}_i) - \mathbf{p}_i \cdot \nabla\mathbf{u} - m_i\mathbf{r}_i \cdot \nabla\mathbf{u} \cdot \nabla\mathbf{u} - \frac{p_s}{Q}\mathbf{p}_i - \frac{p_s}{Q}(m_i\mathbf{r}_i \cdot \nabla\mathbf{u} - m_i\mathbf{U}(\mathbf{r}_i)), \qquad \text{(S1)}$$

$$\dot{s} = \frac{p_s}{Q}, \quad \dot{p}_s = F_s(\mathbf{p}_i), \quad Q = DNk_BT\tau_t^2$$

where $\mathbf{r}_i$, $\mathbf{p}_i$, and $\mathbf{F}_i$ indicate the position, (nominal) peculiar momentum, and force vector of atom i of the mass m_i. The s and p_s are position-like and momentum-like variables, respectively, of the Nosé-Hoover thermostat. The Q represents the thermostat mass parameter, for which D, N, and τ_t refer the dimensionality of the system, the total number of atoms, and the thermostat relaxation time parameter, respectively. The τ_t was set equal to 0.24 ps in all simulations. The $\nabla\mathbf{u}$, homogeneous velocity gradient tensor, is expressed as

$$\nabla\mathbf{u} = \begin{bmatrix} 0 & 0 & 0 \\ \dot{\gamma} & 0 & 0 \\ 0 & 0 & 0 \end{bmatrix} \qquad \text{(S2)}$$

where $\dot{\gamma}$ is the shear rate. The streaming velocity $\mathbf{U}(\mathbf{r}_i)$ at atomic position $\mathbf{r}_i$ was evaluated based on a 5th-order polynomial fit in every MD step throughout the total (bulk plus interfacial) region. The real peculiar momentum $\mathbf{p}_i^{\mathrm{real}}$ of each atom was then calculated by

subtracting the streaming velocity at its position from its laboratory momentum $\mathbf{p}_i' = \mathbf{p}_i + m_i \mathbf{r}_i \cdot \nabla \mathbf{u}$:

$$\mathbf{p}_i^{\text{real}} = \mathbf{p}_i' - m_i \mathbf{U}(\mathbf{r}_i) \tag{S3}$$

The p-SLLOD equations of motion with Nosé–Hoover thermostat for both systems were numerically integrated with the reversible REference System Propagator Algorithm (r-RESPA)[27] in multiple time scale: the long time scale (2.35 fs) for relatively weak forces (nonbonded LJ interactions) and the short scale (0.47 fs) for strong forces (bonded interactions).

In bulk and confined systems, we adopted the Siepmann-Karaboni-Smit (SKS) united-atom model[26] excluding the rigid bond assumption. To assign more flexibility to PE melts, the rigid bond in the original model was substituted with a harmonic potential function. In the SKS model, nonbonded atomic interactions including intra-, intermolecular interaction were modeled by a pairwise 6-12 Lennard-Jones (LJ) potential:

$$U_{\text{LJ}}(r_{ij}) \;=\; 4\varepsilon_{ij}\left[\left(\frac{\sigma_{ij}}{r_{ij}}\right)^{12} - \left(\frac{\sigma_{ij}}{r_{ij}}\right)^{6}\right] \tag{S4}$$

where $\varepsilon_{ij} = (\varepsilon_i \varepsilon_j)^{1/2}$ and $\sigma_{ij} = (\sigma_i + \sigma_j)/2$ were followed the standard Lorentz-Berthelot mixing rules between atomistic units i and j. The LJ energy and length parameters was assigned to ε/k_B = 47 K, σ = 3.93 Å for CH_2 units and ε/k_B = 114 K and σ = 3.93 Å for CH_3 units. The $r_c = 2.5\ \sigma_{ij}$ is a cut-off distance for both atom-atom and atom-wall. In our confined system simulations, wall atoms only interact with fluid atoms, not with each wall atoms. The bonded atomic interactions involving bond-stretching (U_{str}), bond-bending (U_{ben}) and bond-torsional (U_{tor}) were described by

$$U_{\text{str}}(l) = \frac{k_{\text{str}}}{2}(l - l_{\text{eq}})^2 , \quad \text{(S5)}$$

$$U_{\text{ben}}(\theta) = \frac{k_{\text{ben}}}{2}(\theta - \theta_{\text{eq}})^2 , \quad \text{(S6)}$$

$$U_{\text{tor}}(\phi) = \sum_{m=0}^{3} a_m (\cos\phi)^m \quad \text{(S7)}$$

Where, for bond-stretching interaction, the bond-stretching constant is k_{str}/k_B = 452,900 K/Å^2 and the equilibrium bond length l_{eq} = 1.54 Å. For bond-bending interaction, the bond-bending constant is k_{ben}/k_B = 62,500 K/rad^2 and the equilibrium bond angle θ_{eq} = 114°. For bond-torsional interaction, the bond-torsional constants are a_0/k_B = 1010 K, a_1/k_B = 2019 K, a_2/k_B = 136.4 K, and a_3/k_B = −3165 K.

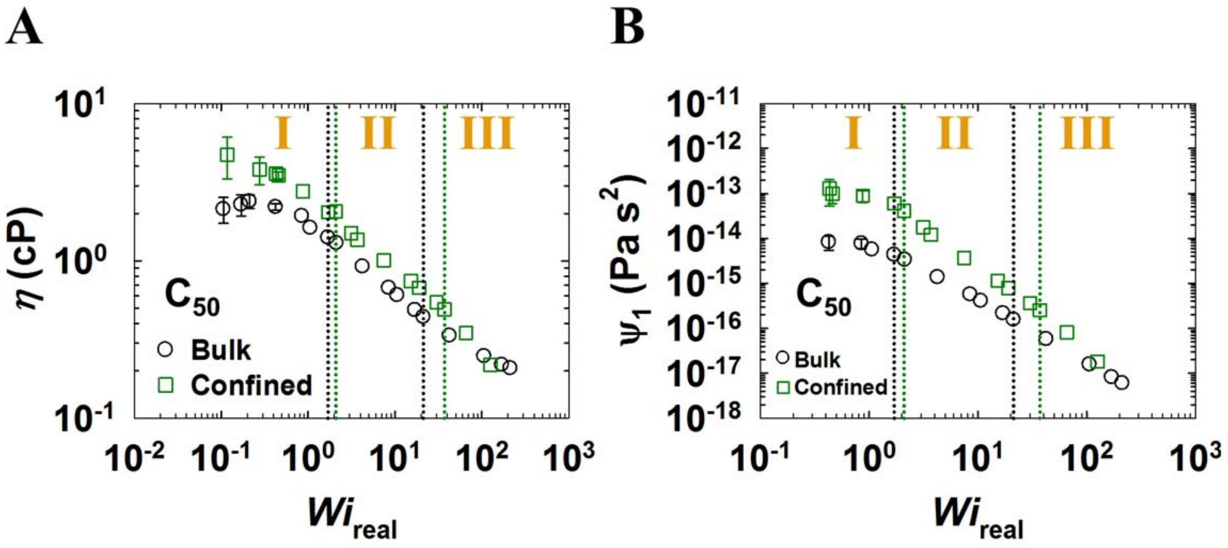

Figure S1. Comparison between bulk and confined systems for (A) shear viscosity η and (B) the first normal stress coefficient (Ψ_1) as a function of Wi_{real} based on the real shear rate accounting for a non-zero slip at the wall for the C_{50} PE melt under shear flow. Note that there is no change in case of the bulk system already applied no-slip boundary condition. The vertical dotted lines distinguish the three flow regimes for each system: Black, bulk system; Green, confined system. The error bars are smaller than the size of the symbols unless otherwise specified.

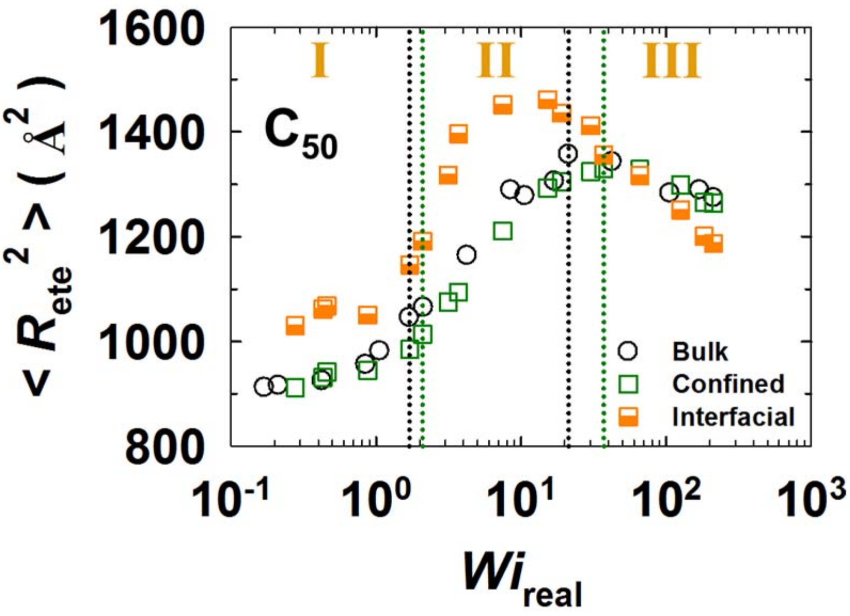

Figure S2. The mean-square chain end-to-end distance $\langle R_{ete}^{2} \rangle$ as a function of Wi_{real} (based on the real shear rate accounting for a non-zero slip at the wall) for the bulk and confined C_{50} PE melt systems. 'Interfacial' represents the corresponding result only for the interfacial chains in the confined system. The vertical dotted lines distinguish the three flow regimes for each system: Black, bulk system; Green, confined system. Note that green dotted lines also correspond to the interfacial region. The error bars are smaller than the size of the symbols unless otherwise specified.

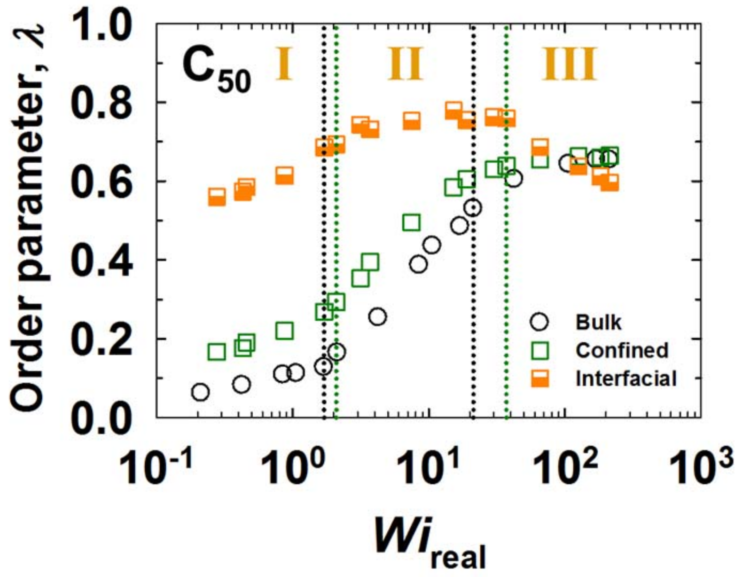

Figure S3. Chain order parameter λ as a function of Wi_{real} (based on the real shear rate accounting for a non-zero slip at the wall) for the bulk and confined C_{50} PE melt systems. 'Interfacial' represents the corresponding result only for the interfacial chains in the confined system. The vertical dotted lines distinguish the three flow regimes for each system: Black, bulk system; Green, confined system. Note that green dotted lines also correspond to the interfacial region. The error bars are smaller than the size of the symbols unless otherwise specified.

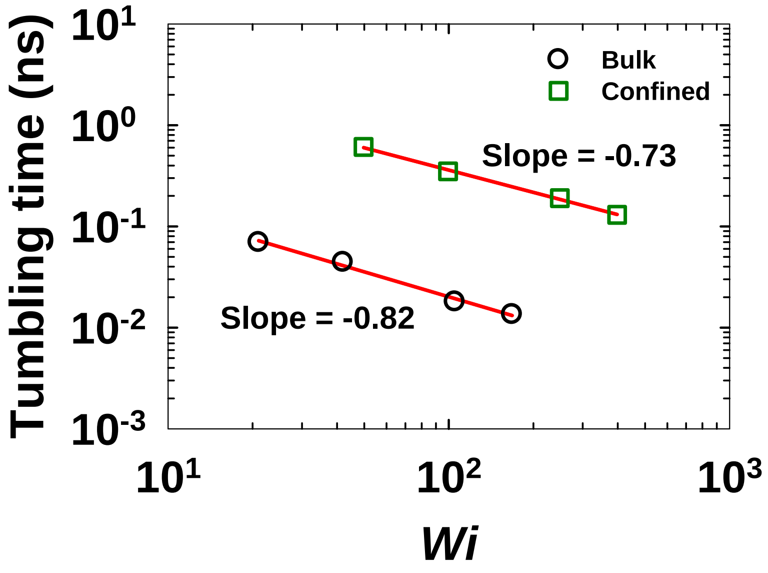

Figure S4. Average tumbling time calculated by measuring the time decay of the correlation function $\left\langle \mathbf{R}_x(t)\mathbf{R}_y(t+\tau)\right\rangle$ for the *x*- and *y*-component of the chain end-to-end vector, **R**, for the bulk and confined C_{50} PE melt systems in the strong flow regime. Because of the relatively slower rotational dynamics of chains near the wall (i.e., the interfacial region), the average tumbling time is larger for the confined system than the corresponding bulk system. (Note that we are not able to calculate the tumbling time only for the interfacial chains due to a frequent exchange of the chains in their locations between the bulk and interfacial region.)
